# Effect of Regulated Add-on Sodium Chloride Intake on Stabilization of Serum Lithium Concentration in Bipolar Disorder: A Randomized Controlled Trial

**DOI:** 10.1192/j.eurpsy.2023.620

**Published:** 2023-07-19

**Authors:** R. Maiti, S. George, B. R. Mishra, M. Jena

**Affiliations:** ^1^Pharmacology; ^2^Psychiatry, All India Institute of Medical Sciences Bhubaneswar, Bhubaneswar, India

## Abstract

**Introduction:**

The therapeutic use of lithium in bipolar disorder is often restricted due to its narrow therapeutic window and adverse drug reactions. Lithium-induced early renal dysfunction is clinically important as it may lead to sodium depletion due to natriuresis leading to lithium retention and lithium toxicity. This is most often seen in the initial phases of therapy, and psychiatrists struggle titrating the dose of lithium and stabilizing the serum lithium level.

**Objectives:**

The present study was conducted to evaluate the effect of add-on sodium chloride on serum lithium levels in bipolar disorder.

**Methods:**

The present randomized controlled trial (NCT04222816) was conducted in 60 patients with type I bipolar disorder who were randomized into the control group who received lithium carbonate with the advice not to take additional salt (at the table) and the test group who received sachets of sodium chloride (1 g/d) as an add-on to lithium carbonate and were advised to restrict their additional salt intake (at the table) to 1 g/d. After baseline assessments, all patients were followed up at 4 weeks, 8 weeks, and 12 weeks when serum lithium, sodium and potassium were estimated. Serum creatinine and aldosterone were repeated at 12 weeks.

**Results:**

In the test group, the fluctuation rate in serum lithium (26.7%) was significantly (p=0.01) lower than in the control group (63.3%). There was a significant difference in serum lithium in the control group at different time points; however, the changes were not significant in the test group. There was a significant difference in serum lithium between the groups at 8 and 12 weeks of follow-up. There were no significant differences in the change in serum sodium, potassium, creatinine, aldosterone, creatinine clearance, and blood pressure within the group and between the groups. A significant positive correlation was found between serum lithium and aldosterone at baseline.

**Conclusions:**

Intake of add-on sodium chloride (1 gm/day) may reduce the fluctuations in serum lithium during the maintenance phase of lithium therapy in type I bipolar disorder.

**Disclosure of Interest:**

None Declared

